# Relationship of prepartum udder and teat measurements with subsequent milk production traits in primiparous Nili-Ravi buffaloes

**DOI:** 10.14202/vetworld.2016.1173-1177

**Published:** 2016-11-02

**Authors:** T. Chandrasekar, Kalyan Sundar Das, Showkat A. Bhat, J. K. Singh, Thulasiraman Parkunanan, K. Puhle Japheth, Mayur R. Thul, Pranay Bharti

**Affiliations:** 1 Department of Livestock Production and Management, ICAR - National Dairy Research Institute, Karnal - 132 001, Haryana, India; 2 ICAR - Agricultural Technology Application Research Institute, Bhumi Vihar Complex, Block - III, Salt Lake City, Kolkata - 700 097, West Bengal, India; 3 ICAR - Central Institute for Research on Cattle, Meerut - 250 001, Uttar Pradesh, India; 4 Division of Cattle Physiology, ICAR - National Dairy Research Institute, Karnal - 132 001, Haryana, India

**Keywords:** buffalo heifers, milk yield, peak yield, primiparous buffalo, teat measurements, udder measurements

## Abstract

**Aim:**

This study was conducted to find out the relationship of prepartum udder and teat measurements with subsequent milk production traits in primiparous Nili-Ravi buffaloes.

**Materials and Methods:**

A total of 12 Nili-Ravi buffalo heifers were selected from Buffalo Farm, Central Institute for Research on Buffaloes, Regional Station- Bir Dosanjh, Nabha, Patiala, Punjab. The udder length (UL), udder width (UW), udder depth, teat length (TL), teat diameter (TD), and teat distances were measured at fortnightly interval from 60 days prepartum until calving. After calving, 60 days total milk yield (TDMY), peak yield (PY), and days taken to attain PY (DPY) were also recorded. The correlation coefficients of various prepartum udder and teat measurements since 60 days prepartum to calving with 60 days TDMY, PY, and DPY were calculated to find out the relationship between the traits in primiparous Nili-Ravi buffaloes.

**Results:**

The result envisaged that all udder and teat measurements were increased gradually toward the date of calving in primiparous buffaloes. The UL, UW, left fore (LF) and right rear (RR)TL, RRTD, and the distance between LF to left rear (LR) teat were positively correlated with 60 days TDMY. The UL and UW depicted positive but nonsignificant correlation with PY. Fore TLs showed positive correlation where as TDs and teat distances had a negative correlation with the DPY in primiparous Nili-Ravi buffaloes.

**Conclusion:**

It was concluded that milk production performance could be assessed on the basis of prepartum udder and teat measurements in primiparous Nili-Ravi buffaloes.

## Introduction

Morphological characteristics of udder and teats have a direct relation with milk production potential in dairy animals. Udder and teat measurements show variation between breeds and individuals in the same herd [1].

This has utmost importance while selecting dairy animals which are to be used for future milk production. The milk production of multiparous animals is generally predicted on the basis of their previous lactation performance. However, in primiparous animals, as the direct performance data are not available, stock persons/livestock owners use to establish the relationships among mammary morphological characteristics and milk production as a tool of selection. Many researchers correlated different udder traits and milk production in multiparous bovines [2-5] and in crossbred primiparous cows [6]. However, the reference in the literature is scanty in this aspect particularly in Nili-Ravi buffaloes.

Hence, our current study intended to find out the correlations of prepartum udder and teat measurements with subsequent milk production traits in primiparous Nili-Ravi buffaloes.

## Materials and Methods

### Ethical approval

This study was conducted after getting approval from the Research Committee and Institutional Animal Ethics Committee of Indian Veterinary Research Institute (IVRI), Izatnagar, Bareilly, Uttar Pradesh.

### Location of the study

This study was conducted at the Buffalo Farm, Central Institute for Research on Buffaloes (CIRBs), Regional Station-Bir Dosanjh, Nabha, Patiala, Punjab during the period September 2013 to March 2014. The farm is situated at an altitude of 250 m above the mean sea level at 30° 22’ 28” N latitude and 76° 8’ 54” E longitude. The ambient temperature of the region generally touches 0°C in winter and 47°C during summer months. Average annual rainfall is about 700 mm, most of which is received mostly during June to September. The Regional Station of CIRBs at Bir Dosanjh, Nabha, Punjab, was established during the year 1987 to conserve and improve this fine breed. This is the only organized pure Nili-Ravi buffalo herd in India.

### Experimental animals

Twelve pregnant Nili-Ravi buffalo heifers were selected from the Buffalo Farm, CIRB, Nabha at 60 days before their expected date of calving. The experimental animals were born between January 30, 2009 and March 01, 2011. The average body weight of pregnant heifers at the start of experiment, i.e., 60 days prepartum was 524 kg which varied from 445 to 612 kg. All the experimental buffaloes were maintained under loose housing and group management system.

### Data recording

The data were recorded at fortnightly interval from 60 days prepartum until calving (i.e., on 60^th^, 45^th^, 30^th^, 15^th^ day before expected date of calving and at calving) for different udder traits, i.e., udder length (UL), udder width (UW), udder depth (UD), and teat traits, i.e., teat length (TL) and teat diameter (TD)- Left fore (LF), left rear (LR), right fore (RF), right rear (RR); and teat distance (DBT)- LF-RF, LR-RR, LF-LR, RF-RR, LF-RR, and RF-LR according to the procedure followed by earlier researcher [6]. After calving, milk production traits, i.e. 60 days (test days) total milk yield (TDMY), peak yield (PY) and days taken to attain PY (DPY) were recorded for all experimental buffaloes.

### Statistical analysis

The mean, standard error and coefficient of variation of various udder and teat traits at different days of pregnancy were calculated. The Pearson’s correlation coefficient of all udder and teat traits at different stages of pregnancy with 60 days TDMY, PY, and DPY were calculated using Statistical Analysis System (SAS, 2011) software programme, version 9.3 [7].

### Results and Discussions

The averages of various udder traits during the last 2 months of gestation in primiparous Nili-Ravi buffaloes are presented in Table-1. The mean UL, UW and UD at 2 months before calving were 32.92, 29.92 and 5.17 cm, respectively. At calving, the respective measurements gradually increased upto 50.42, 46.92, and 10.75 cm. A similar trend was also recorded in crossbred heifers in earlier report [8].

**Table 1:** Mean±SE and CV % of various udder traits in pregnant Nili-Ravi buffalo heifers.

Udder traits (cm)	Days before calving									

	60		45		30		15		0	
				
	Mean±SE	CV (%)	Mean±SE	CV (%)	Mean±SE	CV (%)	Mean±SE	CV (%)	Mean±SE	CV (%)
UL	32.92±0.67	7.03	35.33±0.83	8.13	38.33±0.84	7.57	43.58±0.81	6.45	50.42±1.08	7.44
UW	29.92±1.31	15.11	32.25±1.19	12.76	35.83±1.04	10.02	40.25±1.14	9.78	46.92±1.01	7.46
UD	5.17±0.41	27.16	6.75±0.31	15.63	8.08±0.26	11.14	9.67±0.38	13.48	10.75±0.33	10.59

CV=Coefficient of variation, SE=Standard error, UL=Udder length, UW=Udder width, UD=Udder depth

The average TDMY and coefficient of variation in primiparous Nili-Ravi buffaloes were found to be 519.98±25.32 kg and 16.87%, respectively. The average PY and DPY with coefficient of variation were recorded as 10.35±0.42 kg and 14.01%, 48±2.57 days and 18.72%, respectively.

The correlation between udder traits at different days of pregnancy with TDMY and PY in primiparous Nili-Ravi buffaloes is presented in Table-2. The UL and UW showed positive but non-significant correlation with TDMY. The correlation was comparatively higher at 60 days before calving and low at15 days before calving. The positive correlation among TDMY and UL was also reported by earlier researchers in dairy buffaloes [9-11], in swamp buffaloes [3], in crossbred heifers [8,12], and in dairy cows [11]. As per previous findings, TDMY had positive correlations with UW in dairy buffaloes [4,10,11], in swamp buffaloes [3], in crossbred heifers [8], and in dairy cows [11,13,14]. Toward the end of pregnancy, the correlation of UD and TDMY was found to be either very low or negative. Similar negative correlations between the traits were also reported in earlier literatures [15,16]. Contrary to these reports, a positive correlation between UD and TDMY was found by many researchers [1,3,11,12,14,17].

**Table 2:** Correlation coefficient between udder traits and milk production traits at different days of pregnancy in primiparous Nili-Ravi buffaloes.

Days before calving	Milk production traits	Udder traits		

		UL	UW	UD
60	TDMY	0.41	0.37	0.27
	PY	0.46	0.47	0.23
	DPY	−0.30	0.14	0.07
45	TDMY	0.24	0.33	0.01
	PY	0.28	0.40	−0.07
	DPY	−0.28	0.14	−0.12
30	TDMY	0.30	0.39	0.02
	PY	0.36	0.48	−0.04
	DPY	−0.18	−0.01	−0.41
15	TDMY	0.04	0.13	−0.03
	PY	0.13	0.19	−0.02
	DPY	0.13	0.13	−0.52
0	TDMY	0.23	0.30	0.12
	PY	0.30	0.36	0.11
	DPY	−0.32	−0.30	−0.39

TDMY=Total milk yield, PY=Peak yield, DPY=Days taken to attain PY

The results (Table-2) depicted that UL, as well as UW, had positive but non-significant association with PY from 60 days before calving to calving. The correlation was either low or negative among UD and PY throughout the period. However, the correlation values of all udder traits with PY were not statistically significant. In an earlier report, UL, UW, and UD at all stages of pregnancy were highly (p<0.01) correlated with PY and most of the udder measurements from 6.5 to 8 months of pregnancy showed higher associations with PY [8].

The correlation coefficients among various udder traits at different days before calving and DPY in Nili-Ravi primiparous buffaloes are shown in Table-2. The UL had negative correlation throughout the last 2 months pregnancy except at 15 days prepartum where positive value was recorded as 0.13. The UW was positively correlated with DPY at 60, 45, and 15 days prepartum and negatively correlated at 30 days prepartum at the date of calving. Almost no correlation was found between UD and DPY at 60 days prepartum. After that, there was negative correlation upto the date of parturition. The correlation values among those traits were not statistically significant throughout the period.

The averages of different teat traits with coefficient of variations during the last 60 days of pregnancy in primiparous Nili-Ravi buffaloes are presented in Table-3. As per TL was concerned, LF teat before 2 months of pregnancy was 3.89 cm which increased to 5.13 cm at calving. The respective values for LR, RF and RR teat were recorded as 4.03, 3.93 and 4.13 cm, 5.40, 5.18 and 5.49 cm. All TLs gradually increased from 60 days before calving toward calving. The length of rear teats was comparatively greater than fore teats throughout the experimental period. Considering the TD, the LF teat at 60 days before calving was 1.86 cm which gradually increased upto 2.73 cm at calving. Other TDs, i.e., LR, RF, and RR also showed a similar growing trend toward the end of gestation. The highest TD was noted in LR teat followed by RR. Fore teats were almost similar in diameter throughout the last 2 months of pregnancy. Table-3 showed that at 60 days before calving, front and rear teat distances were 8.97 and 2.99 cm, respectively. The respective distances increased as 14.30 and 6.04 cm at the time of calving. Almost similar increase of teat distance was recorded during the period in LR-RR, LF-LR and RF-RR (3.05, 3.39 and 3.90 cm, respectively), whereas a substantial increase was noted in LF-RF followed by LF-RR.

**Table 3:** Mean±SE and CV % of various teat traits in pregnant Nili-Ravi buffalo heifers.

Teat traits (cm)	Days before calving									

	60		45		30		15		0	
				
	Mean±SE	CV (%)	Mean±SE	CV (%)	Mean±SE	CV (%)	Mean±SE	CV (%)	Mean±SE	CV (%)
TL										
LF	3.89±0.11	9.64	4.08±0.13	10.26	4.31±0.11	8.48	4.62±0.12	9.32	5.13±0.13	8.97
LR	4.03±0.17	14.16	4.20±0.17	13.62	4.48±0.17	13.45	4.87±0.22	15.41	5.40±0.22	14.04
RF	3.93±0.17	14.13	4.06±0.16	13.43	4.23±0.17	13.75	4.53±0.23	17.30	5.18±0.22	14.81
RR	4.13±0.19	16.02	4.30±0.17	13.45	4.57±0.20	14.87	4.93±0.23	16.18	5.49±0.24	15.09
TD										
LF	1.86±0.08	15.81	1.98±0.09	15.92	2.13±0.09	14.46	2.35±0.08	12.37	2.73±0.09	11.62
LR	2.08±0.13	22.05	2.27±0.13	19.31	2.44±0.14	19.33	2.71±0.17	21.21	3.06±0.16	18.15
RF	1.86±0.07	13.66	2.03±0.09	15.35	2.18±0.09	14.69	2.33±0.10	14.66	2.73±0.11	13.65
RR	1.99±0.06	9.92	2.16±0.06	10.35	2.38±0.07	10.41	2.58±0.07	9.60	2.95±0.11	12.56
DBT										
LF-RF	8.97±0.90	34.89	9.89±0.86	30.08	10.79±0.76	24.41	12.43±0.66	18.27	14.30±0.74	17.98
LR-RR	2.99±0.28	32.52	3.39±0.31	31.42	4.05±0.34	29.05	4.60±0.34	25.63	6.04±0.43	24.77
LF-LR	3.57±0.28	27.64	3.88±0.28	24.93	4.78±0.41	29.77	5.72±0.42	25.26	6.96±0.55	27.45
RF-RR	3.78±0.30	27.14	4.10±0.33	27.52	4.28±0.30	23.92	5.04±0.33	22.37	6.68±0.50	26.15
LF-RR	7.42±0.55	25.65	8.000.53	22.55	8.88±0.45	19.01	10.65±0.42	13.64	13.46±0.74	19.01
RF-LR	6.99±0.42	20.96	7.46±0.45	20.73	8.14±0.43	18.42	10.23±0.50	17.01	11.73±0.51	15.01

TL=Teat length, TD=Teat diameter, DBT=Teat distance, LF=Left fore, RF=Right fore, LR=Left rear, LF=Left fore, CV: Coefficient of variation, SE=Standard error

The correlation coefficient between teat traits at different days of pregnancy with TDMY and PY in primiparous Nili-Ravi buffaloes has been presented in Table-4. Considering the TL, LF and RR teat showed comparatively higher correlations than other two teats. The correlations of LF, RF and RR TL with TDMY were high at 60 and 45 days prepartum and gradually decreasing trend was recorded toward calving. Mostly, the TLs showed positive associations throughout the period. However, the values were not statistically significant. The previous observations in Nili-Ravi buffaloes [5], in Holstein heifers [16], and in Karan-Fries cows [18] were also in agreement with the present findings. Some researchers reported that fore TL [8,15,18] and rear TL [7,8,19] were more associated with milk production. Contrary to these findings, negative or no correlation between TL and TDMY was found in some reports [20,21]. The LF and RR teat showed a positive association with PY since 60 days prepartum to calving (Table-4). There was almost no correlation between LR teat and PY. The RF teat was positively correlated with PY at 60 and 45 days prepartum which gradually decreased at 15 days prepartum. Finally, at calving, no/very low negative correlation was established. However, no reference in the literature was available to compare this study.

**Table 4:** Correlation coefficients of teat traits with TDMY and PY at different days of pregnancy in primiparous Nili-Ravi buffaloes.

Teat traits (cm)	Days before calving									

	60		45		30		15		0	
				
	TDMY	PY	TDMY	PY	TDMY	PY	TDMY	PY	TDMY	PY
TL										
LF	0.41	0.31	0.45	0.31	0.37	0.28	0.31	0.20	0.12	0.07
LR	0.05	−0.02	0.10	−0.01	0.19	0.08	0.15	0.06	0.12	−0.01
RF	0.28	0.21	0.30	0.21	0.24	0.12	0.18	0.10	0.07	−0.03
RR	0.38	0.31	0.33	0.26	0.41	0.31	0.37	0.27	0.21	0.12
TD										
LF	−0.17	−0.22	−0.04	−0.14	−0.16	−0.22	−0.41	−0.44	−0.23	−0.24
LR	0.26	0.18	0.20	0.09	0.15	0.10	0.09	0.03	0.06	0.02
RF	0.00	−0.09	0.09	−0.03	−0.09	−0.16	−0.05	−0.12	0.15	−0.02
RR	0.10	0.06	0.20	0.07	0.19	0.21	0.34	0.32	0.59*	0.47
DBT										
LF-RF	0.10	0.22	−0.10	0.04	−0.13	−0.03	−0.14	−0.09	−0.02	0.05
LR-RR	−0.55	−0.51	−0.70	−0.66	−0.54	−0.45	−0.57	−0.47	−0.28	−0.23
LF-LR	0.37	0.48	0.26	0.38	0.20	0.39	0.19	0.34	0.27	0.38
RF-RR	0.02	0.20	−0.18	0.01	0.03	0.21	0.12	0.30	0.19	0.30
LF-RR	0.04	0.09	0.03	0.04	−0.01	0.07	0.20	0.37	0.25	0.32
RF-LR	−0.06	0.01	−0.18	−0.12	−0.19	−0.11	0.04	0.16	−0.08	0.04

TL=Teat length, TD=Teat diameter, DBT=Teat distance, LF=Left fore, RF=Right fore, LR=Left rear, LF=Left fore, TDMY=Total milk yield, PY=Peak yield, DPY=Days taken to attain peak yield, RR=Right rear

Either very low positive or negative correlation was found between TDMY and fore TD throughout the last 2 months of gestation (Table-4). Rear TD showed a positive association with the TDMY. The relationship between RR TD and TDMY was found significant (0.05%) at calving. Earlier reports also indicated positive [5,8,16,17] and significant [4-6,17] correlations among TDs and TDMY. It was highlighted that one cm increase of front TD resulted 151.5 g increase in milk yield [17]. On the other hand, some negative correlations among traits were also available in the reports [18,21].

Fore TD had negative association with PY throughout the last months of gestation. The correlation values of LR TD and PY were positive and highest at 60 days prepartum which gradually decreased toward the end of gestation. The opposite trend was found in the case of RR TD where the highest correlation was noted at calving.

Table-4 envisaged that LF-LR was positively associated with TDMY during the last 2 months of gestation. The negative correlation was recorded between LR-RR and TDMY. All other teat distances, i.e., LF-RF, RF-RR, LF-RR, and RF-LR showed either positive or correlation with the trait. Milk production, significantly correlated with teat distance, was also found by many researchers in their studies [6,16]. Some of the reports also pointed out that fore teatdistance [1,15,19], rear teat distance [1,15] LF-LR distance [1,8,14], and RF-RR distance [1,14] were most important for milk production point of view.

The lateral teat distances were positively correlated with PY during the study period. Rear teat distance for the last 2 months of pregnancy had a high negative correlation with PY. Positive and high correlation between LF-RR and PY was observed during the last 15 days of calving. The RF-LR and LF-RF showed either low positive or negative correlation. The correlation values were not statistically significant. These findings were partially in agreement with the previous findings [8,19].

The correlation coefficient between various teat traits at different days of pregnancy and DPY in primiparous Nili-Ravi buffaloes is presented in Table-5. The correlation values of fore TLand DPY were positive throughout the experimental period. The values gradually decreased toward the calving. Rear TL had either very low or no correlation with DPY. The correlation of LF and LR TD with DPY was gradually decreased from 60 days prepartum to calving. The RF and RRTDs showed negative correlation without any particular trend (Table-5).

**Table 5:** Correlation coefficients between teat traits and DPY at different days of pregnancy in primiparous Nili-Ravi buffaloes.

Teat traits (cm)	Days before calving				

	60	45	30	15	0
TL					
LF	0.44	0.53	0.26	0.16	0.03
LR	0.08	0.09	0.00	−0.02	0.09
RF	0.35	0.36	0.30	0.23	0.25
RR	0.13	0.16	0.07	−0.02	−0.12
TD					
LF	−0.04	0.00	−0.25	−0.28	−0.39
LR	−0.05	−0.06	−0.33	−0.45	−0.47
RF	−0.22	−0.08	−0.37	−0.38	−0.11
RR	−0.05	0.01	−0.59	−0.40	−0.09
DBT					
LF-RF	−0.21	−0.36	−0.51	−0.45	−0.72
LR-RR	0.04	−0.08	−0.39	−0.43	−0.58
LF-LR	0.18	0.10	−0.26	−0.06	−0.07
RF-RR	−0.32	−0.40	−0.32	−0.02	0.08
LF-RR	−0.18	−0.26	−0.48	−0.67	−0.50
RF-LR	−0.12	−0.08	−0.25	−0.32	−0.44

TL=Teat length, TD=Teat diameter, DBT=Teat distance, LF=Left fore, RF=Right fore, LR=Left rear, LF=Left fore, RR=Right rear, DPY=Days taken to attain peak yield

The result depicted that all teat distances established negative correlation with DPY throughout the last 2 months of gestation in Nili-Ravi buffaloes. Although some values were high, they were not statistically significant.

## Conclusion

This study revealed that udder and teat measurements particularly UL, UW, LF and RRTL, RRTD, and distance between LF to LR teat were positively associated with subsequent milk production. The UL and UW depicted positive and non-significant correlation with PY. Fore TLs showed positive correlation whereas TDs as well as distances had a negative correlation with DPY. Therefore, it can be concluded that udder and teat measurements in pregnant Nili-Ravi buffalo heifers are useful for assessing the subsequent milk production performance.

## Authors’ Contributions

TC, KSD, and JKS designed the work. TC conducted the research work. Data analysis and manuscript was written by SAB, TP, KPJ, MRT, and PB under the guidance of KSD and JKS. All the authors have read and approved the final manuscript.
